# Exercise and gastrointestinal symptoms: running-induced changes in intestinal permeability and markers of gastrointestinal function in asymptomatic and symptomatic runners

**DOI:** 10.1007/s00421-017-3739-1

**Published:** 2017-10-14

**Authors:** Elisa Karhu, Richard A. Forsgård, Lauri Alanko, Henrik Alfthan, Pirkko Pussinen, Esa Hämäläinen, Riitta Korpela

**Affiliations:** 1 Pharmacology, University of Helsinki, P.O. Box 63, 00014 Helsinki, Finland; 2 Clinic for Sports and Exercise Medicine, Foundation for Sport and Exercise Medicine, Helsinki, Finland; 3 HUSLAB, Helsinki University Hospital, Helsinki, Finland; 4 Department of Clinical Chemistry, University of Helsinki, Helsinki, Finland; 5 Oral and Maxillofacial Diseases, University of Helsinki and University Hospital of Helsinki, Helsinki, Finland

**Keywords:** Intestinal permeability, Exercise, Gastrointestinal symptoms, I-FABP, LPS

## Abstract

**Purpose:**

Athletes frequently experience gastrointestinal (GI) symptoms during training and competition. Although the prevalence of exercise-induced GI symptoms is high, the mechanisms leading to GI distress during exercise are not fully understood. The aim of this study was to identify running-induced changes in intestinal permeability and markers of GI function and investigate their association with gastrointestinal symptoms.

**Methods:**

We recruited 17 active runners who we allocated as either asymptomatic or symptomatic based on their history of experiencing GI symptoms during running. The participants took part in a running test where they were asked to run for 90 min at 80% of their best 10 km race speed. Intestinal permeability was measured at baseline and after the running test. Levels of serum intestinal fatty acid-binding protein (I-FABP), zonulin, bacterial lipopolysaccharide (LPS), and fecal calprotectin were also measured at baseline and after the running test.

**Results:**

Running induced a significant increase in intestinal permeability and serum I-FABP concentration but there were no differences between asymptomatic and symptomatic runners. Serum LPS activity did not change from baseline following the running test but the symptomatic group exhibited higher LPS activity at baseline compared to the asymptomatic runners.

**Conclusions:**

Running for 90 min at a challenging pace causes small intestinal damage and increases intestinal permeability. However, these alterations in GI function do not appear to correlate with the development of GI symptoms during running.

## Introduction

Exercise-induced gastrointestinal (GI) symptoms such as diarrhea, cramping, nausea and gastric pain occur frequently in runners during training and competitions. The prevalence of GI symptoms during exercise varies across studies with estimates ranging from 30 to up to 90% depending on the study design (de Oliveira et al. 2014). In addition to the high prevalence of these symptoms, exercise-induced GI distress may negatively impact athletic performance and in some cases, lead to dropping out of the competition (Hoffman and Fogard 2011; de Oliveira et al. 2014). However, the mechanisms leading to GI distress are not fully understood, nor the reasons why some remain asymptomatic.

Exercise affects GI function via several mechanisms that may give rise to exercise-induced GI disturbances. Although the etiology of exercise-induced GI disturbances involves multiple physiological and pathophysiological mechanisms (de Oliveira et al. 2014), studies have suggested that the key culprit behind GI symptoms during exercise is splanchnic hypoperfusion (ter Steege and Kolkman 2012; van Wijck et al. 2012). Splanchnic hypoperfusion during exercise may lead to intestinal ischemia that subsequently damages intestinal epithelial cells and compromises the intestinal barrier function. So far, multiple studies have reported exercise-induced increases in intestinal permeability (Oktedalen et al. 1992; Pals et al. 1997; van Nieuwenhoven et al. 2004; Marchbank et al. 2011; van Wijck et al. 2011; Zuhl et al. 2015; Davison et al. 2016), serum endotoxemia (Bosenberg et al. 1988; Jeukendrup et al. 2000; Ashton et al. 2003), and inflammatory markers (Jeukendrup et al. 2000; Gill et al. 2015) but whether these changes are due to splanchnic hypoperfusion is still unclear. Also, contradictory findings exist (Ryan et al. 1996; van Nieuwenhoven et al. 1999; Van Wijck et al. 2012) and the association between impaired barrier function and GI symptoms during exercise remains poorly characterized.

The aim of this study was to measure running-induced changes in intestinal permeability and markers of GI function and investigate their association with gastrointestinal symptoms. Intestinal permeability was assessed via oral administration of iohexol, a 821 Da sized contrast agent that has proved to be a reliable marker of intestinal permeability (Halme et al. 1993, 2000; Frias et al. 2014; Forsgård et al. 2016). We also measured serum concentrations of intestinal fatty acid-binding protein (I-FABP), a marker of enterocyte damage (van Wijck et al. 2011) and zonulin, an endogenous protein that specifically and reversibly regulates intestinal permeability (Fasano 2012). In addition, we measured serum bacterial lipopolysaccharide (LPS) activity and analyzed the level of intestinal inflammation by measuring fecal calprotectin concentrations. To our knowledge, this is the first study to directly compare alterations in intestinal permeability in asymptomatic and symptomatic runners.

## Materials and methods

### Ethical statement

The study was conducted in adherence to the ethical regulations outlined in the Declaration of Helsinki and the study was approved by the Hospital District of Helsinki and Uusimaa (HUS) Coordinating ethics committee (13/13/03/00/2015). Informed consent was obtained from all individual participants included in the study.

### Participants

**Fig. 1:**
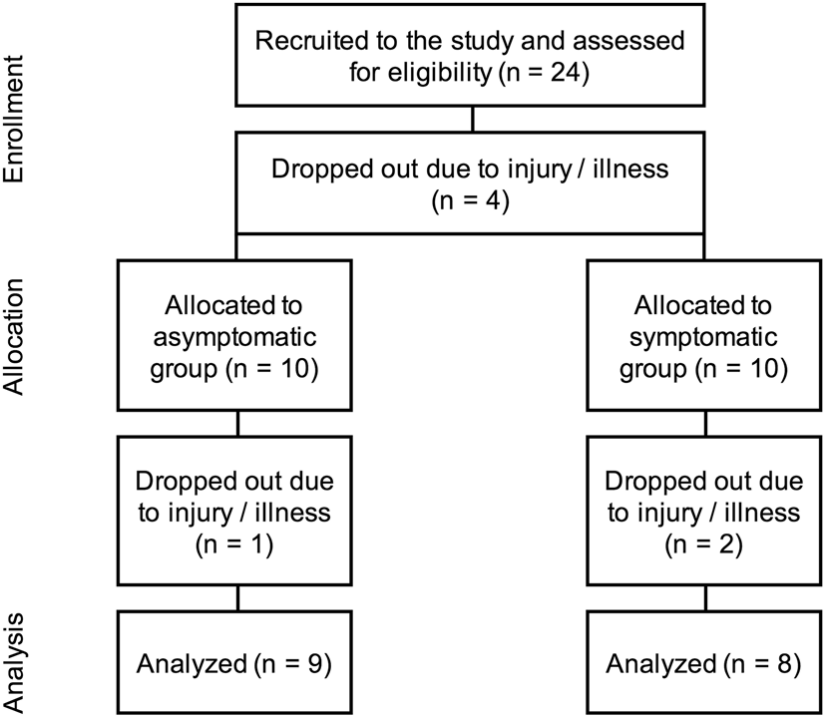
Flowchart showing the number of study participants in each step from enrollment to final analyses

Study participants were recruited by a recruitment notice posted in online groups for active runners around the Helsinki area. Voluntary participants received an informational letter about the study, a consent form, and a symptom history questionnaire used to determine the participant’s suitability for the study. Suitable participants were active runners between the age of 18 and 45 who reported to complete at least three long-distance runs per week. Consenting volunteers suitable to the study were accepted as participants. A total of 24 participants were recruited of which 7 dropped out due to injury or illness (Fig. 1). Persons with a diagnosed gastrointestinal illness or asthma, heart, or cardiovascular diseases were excluded from participation. Pregnant or breastfeeding women were also excluded as well as persons with iodine allergy because of the iohexol used in determining permeability.

### Study protocol

The study participants were first asked to fill out a symptom questionnaire (21 items) that probed the nature and frequency of their GI symptoms during running. Based on their answers, the participants were allocated into two groups: asymptomatic and symptomatic. Participants were deemed symptomatic if they reported having GI symptoms at least 50% of their runs and asymptomatic if they reported experiencing GI symptoms in less than 10% of their runs. Baseline samples at rest were collected 3 weeks before the running test. On the running test day, intestinal permeability measurement (measurement of intestinal permeability) was started before the running test where the participants ran for 90 min at 80% of their best 10 km race speed (running test). A blood sample was withdrawn immediately after the run and the participants were asked to collect a fecal sample at home. The subjects also received a symptom questionnaire (eight items) where they were asked to score on a visual analog scale (VAS) how much stomach pain they experienced during the run. The occurrence of other GI symptoms was also asked in the symptom questionnaire. The participants were also given a questionnaire (11 items) where they were asked to record all GI symptoms they experience later that day. The participants were also instructed to keep a food diary for 3 days before the baseline measurements and the running test day.

### Running test

The subjects ran for 90 min at a challenging pace which was suggested as 80% of the speed of their best 10 km race time. The effort should have been challenging, but able to be maintained for the full 90 min. The running pace was determined individually by the athlete according to their perceived exertion.

### Blood sampling

A blood sample was taken at baseline at rest and then immediately after the running test. The blood sample volume was approximately 5 ml. The blood samples were collected in serum separation tubes [VenoSafe™ Clot Act. (Z), Terumo Europe, Leuven, Belgium] from the median cubital vein by a licensed physician. Serum was separated by centrifugation (1500*g*, 10 min), collected and stored at − 80 °C for later analysis.

### Fecal sample

The study subjects were asked to provide a fecal sample at rest 3 weeks before the running test and then after the running test. The subjects were given written instructions and they collected the fecal samples independently at home in a provided specimen container stored at room temperature. The samples were returned the next day and stored at − 20 °C within the same day for later analysis.

### Measurement of intestinal permeability

Intestinal permeability was determined through oral administration of 50 ml 755 mg iohexol/ml solution (Omnipaque 350™, GE Healthcare, Oslo, Norway) according to a previously published method (Halme et al. 2000). Briefly, the subjects ingested the iohexol solution dissolved in tap water after which they collected all excreted urine in a specimen container for 24 h. The iohexol concentrations in the urine were measured using the iohexol enzyme-linked immunosorbent assay (ELISA) kit (BioPAL, Worcester, MA, USA) according to the kit instructions. The intra-assay coefficient of variation (CV%) of this assay was 4.8% and the limit of detection 0.05 μg/ml. Sample analysis was conducted on the same microtiter plate to decrease inter-assay variation.

### Measurement of serum I-FABP

Serum I-FABP concentrations were measured using I-FABP ELISA test kit (Hycult Biotech, Uden, The Netherlands) per the kit instructions. The intra-assay CV% of this analysis was 5.2% with a detection limit of 25 pg/ml. Sample analysis was conducted on the same microtiter plate to decrease inter-assay variation.

### Measurement of serum zonulin

Serum zonulin concentrations were measured using Zonulin ELISA test kit (Immunodiagnostik, Bensheim, Germany) according to the kit instructions. The intra-assay CV% was 4.6% and the limit of detection 0.225 ng/ml. Sample analysis was conducted on the same microtiter plate to decrease inter-assay variation.

### Measurement of serum bacterial lipopolysaccharide

Serum activity of bacterial lipopolysaccharide (LPS) was analyzed using LAL (Limulus Amebocyte Lysate) Chromogenic Endpoint Assay (Hycult biotech, Uden, The Netherlands) according to the kit instructions. The intra-assay CV% of the assay is 1.9% and the limit of detection 0.04 EU/ml. Sample analysis was conducted on the same microtiter plate to decrease inter-assay variation.

### Measurement of fecal calprotectin

Fecal calprotectin concentrations were measured using Calpro ELISA test (Calpro AS, Oslo Norway) per the kit instructions and a BEP 2000 advance ELISA analyzer (Siemens Healthcare Diagnostics, Munich, Germany). The detection limit of the Calpro ELISA assay in our laboratory is 5 μg/g with an intra-assay CV% below 4.6%. All samples were analyzed in the same batch and on the same microtiter plate to decrease inter-assay variation.

### Food diary

The participants were asked to keep a food diary for 3 days before the baseline measurements and the running tests. The participants were provided with detailed instructions for completion of the food diary. Food diaries were analyzed using Fineli Ruokakori (Foodbasket) database (http://www.fineli.fi/foodbasket.php) for average intake of macronutrients and selected micronutrients over each 3-day food diary period. Analysis of the food diary revealed no statistically significant differences in the average intake of various nutrients from baseline to the running test day or between the groups at these time points (data not shown).

### Data analysis

Normality of the data sets was tested with Kolmogorov–Smirnov test and based on these analyses, differences between the symptomatic and the asymptomatic groups were analyzed using independent samples *t* test. Changes within groups from baseline to after the running test measurements were analyzed using paired samples *t* test. All data are expressed as means ± standard deviations. Statistical calculations were made by PASW Statistics software version 18.0.2. (IBM, Armonk, NY, USA). Figures were created with GraphPad Prism 5 (GraphPad Software Incorporated, La Jolla, CA, USA). Data were deemed significant when *p* < 0.05.

## Results

### Participants

The participants were 24- to 44-year-old (avg. 32.6 ± 6.5) active male and female runners. The asymptomatic group consisted of nine runners (five males and four females) and the symptomatic group of eight runners (four males and four females). Overall, the symptomatic group reported to experience significantly (*p* < 0.001) more GI symptoms during running than the asymptomatic group. From the individual GI symptoms, flatulence (*p* < 0.05) and diarrhea (*p* < 0.001) were significantly more frequent during running in the symptomatic group than in the asymptomatic group. The baseline characteristics of the participants are listed in Table 1. In the symptom history questionnaire, 8 of the 17 runners reported that they usually have loose stool or diarrhea after running. Two reports were from the asymptomatic group and six from the symptomatic group. The remaining seven runners in the asymptomatic group all reported to have normal stool consistency after running.

**Table 1:** Baseline characteristics of participants

	Asymptomatic	Symptomatic	*p*
*n*	9	8	
Males	5	4	
Females	4	4	
Age	34.6 ± 6.1	30.4 ± 6.6	0.196
Symptom frequency	1.9 ± 0.33	3.9 ± 0.64	< 0.001
Frequency of individual symptoms			
Stomach pain	1.8 ± 0.44	2.3 ± 1.0	0.261
Nausea	1.6 ± 0.53	2.1 ± 0.83	0.109
Throwing up	1.3 ± 0.50	1.5 ± 0.53	0.517
Bloating	1.9 ± 0.78	2.4 ± 1.1	0.295
Burping	1.6 ± 0.73	2.3 ± 1.0	0.127
Flatulence	2.1 ± 0.93	3.5 ± 1.1	0.012
Heartburn	1.4 ± 0.73	2.0 ± 1.2	0.259
Liquid in stomach	1.9 ± 0.33	2.5 ± 0.53	0.017
Diarrhea	1.6 ± 0.53	3.8 ± 0.89	< 0.001
Constipation	1.3 ± 0.50	1.3 ± 0.46	0.728
Sum of symptom scores	18.3 ± 3.7	27.4 ± 4.3	< 0.001

1—never experience symptoms; 2—rarely experience symptoms (< 10% of runs); 3—sometimes experience symptoms (50% of runs); 4—often experience symptoms (> 50% of runs); 5—always experience symptoms (> 90% of runs); age and symptom frequencies are listed as means ± standard deviations

### Running test

All participants were able to complete the running test. The average pace was 5:09 ± 0:48 min/km with no difference in pace between the groups. A total of five participants in the symptomatic group and three participants in the asymptomatic group reported to have experienced at least some degree of stomach pain during the running test. On the VAS, there was a non-significant trend for higher average stomach pain in the symptomatic group than in the asymptomatic group (2.0 ± 2.1 vs. 0.30 ± 0.56, *p* = 0.061). All participants in the symptomatic group and five participants in the asymptomatic group reported to have had at least one GI symptom during or following the running test. The symptom results of the running test are summarized in Table 2. Stool consistency after the run was loose in 10 of the 17 runners, with 5 reports from each group. The symptomatic group also reported two counts of hardened stools and one count of diarrhea. The asymptomatic group reported three counts of normal stool and one count of hardened stool.

**Table 2:** Pace, stomach pain, and symptom frequency in the two groups during and following the running test

	Asymptomatic	Symptomatic	*p*
*n*	9	8	
Pace (min/km)	5:12 ± 0:59	5:06 ± 0:39	0.821
Stomach pain (VAS, cm)	0.30 ± 0.56	2.0 ± 2.1	0.061
Frequency of individual symptoms			
Nausea	0/9	3/8	
Throwing up	0/9	0/8	
Bloating	0/9	2/8	
Burping	1/9	3/8	
Flatulence	4/9	4/8	
Heartburn	0/9	1/8	
Liquid in stomach	0/9	1/8	
Diarrhea	1/9	3/8	
Constipation	1/9	2/8	
Subjects that reported at least one GI symptom	5/9	8/8	

Pace and stomach pain listed as means ± standard deviations, individual GI symptoms are reported as the number of study subjects in the group reporting such symptom

### Intestinal permeability

**Fig. 2:**
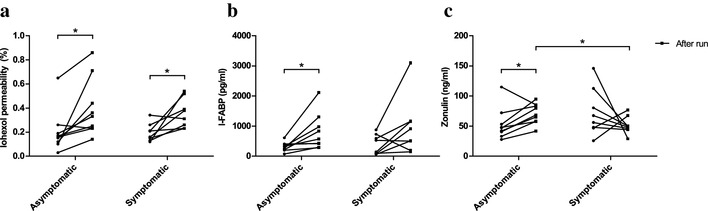
Running induced a significant increase in intestinal permeability to iohexol in both study groups (**a**). Running also increased serum I-FABP concentrations but this increase reached statistical significance only in the asymptomatic group (**b**). Serum zonulin increased significantly in the asymptomatic group following the run (**c**). Circles represent baseline values with lines indicating the change from baseline to after run values. *n* = 9 in the asymptomatic group in all, *n* = 8 in the symptomatic group in all. *p < 0.05

Intestinal permeability increased significantly (*p* < 0.05) in both study groups from baseline to after run measurement (Fig. 2a). The mean iohexol permeability in the asymptomatic group was 0.20 ± 0.18% at baseline and 0.39 ± 0.24% after running. In the symptomatic group, the mean iohexol concentration was 0.20 ± 0.07% at baseline and 0.36 ± 0.12% after running. There was no significant difference in the increase in permeability between the symptomatic and asymptomatic group.

### Serum I-FABP

In the asymptomatic group, serum I-FABP concentrations were significantly (*p* < 0.05) higher following the running test (804 ± 599 pg/ml) compared to the baseline values (314 ± 152 pg/ml) (Fig. 2b). In the symptomatic group, there was a trend for higher serum I-FABP concentrations after running but it did not reach statistical significance (baseline: 389 ± 327 pg/ml; after run: 961 ± 949 pg/ml). There were no significant differences in serum I-FABP concentrations between the groups.

### Serum zonulin

Running induced a significant increase in serum zonulin concentrations in the asymptomatic group (baseline: 53.2 ± 26.3 ng/ml; after run: 70.4 ± 16.7 ng/ml; *p* < 0.05) (Fig. 2c). Serum zonulin concentrations were also significantly (*p* < 0.05) higher in the asymptomatic group than in the symptomatic group (51.3 ± 14.6 ng/ml) following the run (Fig. 2c). There were no significant differences in baseline zonulin concentrations between the groups.

### Serum LPS

**Fig. 3:**
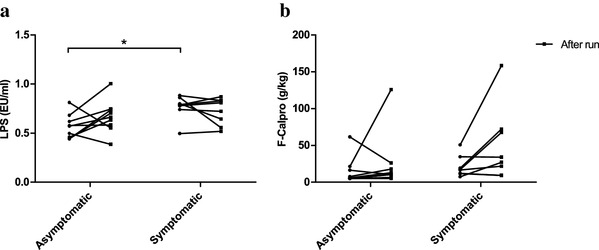
Running did not increase serum LPS (**a**) or fecal calprotectin concentrations (**b**) from baseline. However, the symptomatic group exhibited significantly higher serum LPS concentrations at baseline than the asymptomatic group (**a**). There was also a trend for increased fecal calprotectin concentrations after running in the symptomatic group (**b**). Circles represent baseline values with lines indicating the change from baseline to after run values. *n* = 9 in the asymptomatic group in all, *n* = 8 in the symptomatic group in all. *p < 0.05

Serum LPS activity did not increase from baseline after the running test (Fig. 3a). Serum LPS activity was significantly (*p* < 0.01) higher at baseline in the symptomatic group (0.767 ± 0.119 EU/ml) than in the asymptomatic group (0.567 ± 0.124 EU/ml) (Fig. 3a). There was no significant difference in serum LPS activity between the groups after the run.

### Fecal calprotectin

There were no significant differences in fecal calprotectin concentrations between the groups at baseline or after the run (Fig. 3b). In the asymptomatic group, there were four samples in total (three from baseline, one after run) where calprotectin concentrations were below the assay detection limit (< 5 g/kg) and thus were assigned the value 5 g/kg.

## Discussion

The primary aim of this study was to measure running-induced changes in intestinal permeability and examine their possible association with running-induced GI symptoms. Our results show that running induces a significant increase in urinary iohexol excretion for 24 h indicating increased intestinal permeability. This finding adds to the current literature that has shown mixed results when investigating exercise-induced changes in intestinal permeability (Ryan et al. 1996; Pals et al. 1997; van Nieuwenhoven et al. 1999; Marchbank et al. 2011; van Wijck et al. 2011; Zuhl et al. 2015; Davison et al. 2016; JanssenDuijghuijsen et al. 2016). However, we did not observe any differences in intestinal permeability between asymptomatic and symptomatic runners. To our knowledge, this is the first study that directly compares exercise-induced alterations in intestinal permeability in asymptomatic and symptomatic runners. Previously, van Nieuwenhoven et al. (2004) showed that symptomatic athletes exhibit significantly higher intestinal permeability after cycling than asymptomatic athletes but this comparison was made based on historical asymptomatic controls. Overall, the literature regarding the relationship between exercise-induced GI symptoms and increased intestinal permeability is scarce. Most studies that have directly measured exercise-induced changes in intestinal permeability do not report the occurrence of GI symptoms during exercise or vice versa. This is probably due to difficulties in accurately characterizing and measuring individual GI symptoms during different exercise intensities and durations. This was certainly a limitation in our study as well. Although we achieved a good separation between the two groups at baseline, we observed no clear differences in the occurrence of GI symptoms during or after the running test between the groups. This discrepancy could be due to several factors. First, the subjects in the asymptomatic group reported stomach pain, other GI symptoms, and changes in stool consistency at a higher frequency than expected based on their background questionnaire. In contrast, three symptomatic athletes did not experience any stomach pain during the running test. These findings highlight the difficulties in assigning athletes solely as either asymptomatic or symptomatic and assessing the occurrence of GI symptoms. Second, although suggested to run at challenging pace, the athletes determined their running pace themselves which might have led to variation in individual running intensities and subsequently affected the occurrence of GI symptoms. Third, excluding stomach pain, we gathered data mainly on symptom occurrence so we could not detect any possible differences in symptom severity between the groups. Nonetheless, considering that all but two study subjects exhibited increased intestinal permeability after running, our results imply that increase in intestinal permeability is a normal response to running and it does not appear to correlate with the occurrence of GI symptoms.

Mature enterocytes express I-FABP and upon cellular damage, they release I-FABP into circulation (Adriaanse et al. 2013). Previous studies have shown that the extent of splanchnic hypoperfusion during exercise correlates with serum I-FABP concentrations (van Wijck et al. 2011) and that the small intestine is more prone to intestinal ischemia than the colon (Hundscheid et al. 2015). Together these findings suggest that in our study, the elevated serum concentrations of I-FABP after the running test stem from exercise-induced intestinal ischemia. Whether this explains the observed increase in intestinal permeability is an interesting question. It is important to note that we measured intestinal permeability with a single 24-h urine collection and thus cannot distinguish between small intestinal and colonic permeability. However, Halme et al. (2000) have previously shown that iohexol absorption is constant along the intestinal tract with equal amounts of iohexol present in two 12-h urine fractions. Additionally, other studies that have reported increased intestinal permeability after exercise have employed measurement times ranging from 1 to 5 h (Pals et al. 1997; Marchbank et al. 2011; van Wijck et al. 2011; Zuhl et al. 2015; Davison et al. 2016; JanssenDuijghuijsen et al. 2016) suggesting increased small intestinal permeability. Thus, it is likely that the observed increase in intestinal permeability is a result of running-induced small intestinal damage.

Conceivably, small intestinal damage and increased intestinal permeability could lead to elevated blood LPS activity. Indeed, several studies have reported elevated blood LPS activity after exercise (Brock-Utne et al. 1988; Bosenberg et al. 1988; Jeukendrup et al. 2000; Ashton et al. 2003; Moncada-Jimènez et al. 2009; Lim et al. 2009; Gill et al. 2015), but whether this increase is the result of compromised intestinal barrier function is unclear. In our study, running-induced increase in intestinal permeability did not result in increased serum LPS activity. This finding is similar to Yeh et al. (2013) who showed that running in cool temperature (22 °C) increases intestinal permeability (measured as plasma concentrations of tight junction protein claudin-3) but does not elevate plasma LPS concentrations. These findings suggest that increased intestinal permeability alone does not contribute to exercise-induced increase in blood LPS activity.

Whether elevated blood LPS activity contributes to the development of GI symptoms during exercise is still uncertain. Previously, Brock-Utne et al. (1988) made an intriguing discovery when they reported that ultramarathon runners who had low or normal plasma LPS levels after running experienced less GI symptoms and recovered quicker than runners with high LPS values. However, subsequent studies have not been able to establish a clear correlation between serum LPS levels and GI symptoms during exercise (Jeukendrup et al. 2000; Moncada-Jimènez et al. 2009; Gill et al. 2015). Interestingly, we found that the symptomatic runners had higher serum LPS activity at rest than the asymptomatic runners. This finding raises the question whether some underlying cause, such as nutrition (Kallio et al. 2015) or inadequate adaptation mechanisms to training (Bosenberg et al. 1988; Lim et al. 2009), leads to higher serum LPS activity at rest in symptomatic athletes and subsequently makes them susceptible to GI symptoms during exercise. Overall, the question regarding the relationship between compromised intestinal barrier, blood LPS activity, and GI symptoms during exercise remains open and warrants more research.

Exercise-induced intestinal damage and increased LPS leakage into circulation could trigger an inflammatory response. Multiple studies have shown that fecal calprotectin acts as a reliable marker of inflammation or damage in the gastrointestinal tract (Konikoff and Denson 2006). Our results show a non-significant trend for higher fecal calprotectin concentrations after running suggesting possible activation of inflammatory cascades in the intestinal mucosa. Van Wijck et al. (2011) findings support this conclusion as they showed previously that fecal calprotectin concentrations rise after 60 min of cycling at high intensity. Interestingly, although there was not a significant difference, the average fecal calprotectin after running was twice as high in the symptomatic group as in the asymptomatic group. This is an interesting trend and taken together with previous reports that have described a correlation with increased pro-inflammatory cytokines and GI symptoms during exercise (Jeukendrup et al. 2000; Gill et al. 2015), highlights the possible role of inflammation in mediating some of the detrimental effects of exercise on the GI tract.

We also examined the serum concentration of zonulin, an endogenous protein known to regulate intestinal permeability. The asymptomatic group exhibited significantly elevated serum zonulin levels after running compared to the symptomatic group but the meaning of this finding is difficult to interpret. To our knowledge, only one study has previously examined the effects of exercise on serum zonulin concentrations and they reported no changes after high-intensity interval cycling (JanssenDuijghuijsen et al. 2016).

In conclusion, running for 90 min at a challenging pace induced a significant increase in intestinal permeability. However, we did not observe any differences between asymptomatic and symptomatic runners. The lack of difference in GI symptom occurrence during running between the study groups emphasizes the difficulty of assessing exercise-induced GI symptoms and the need for more robust methods to identify asymptomatic and symptomatic athletes. Nevertheless, the fact that almost all studied athletes exhibited increased intestinal permeability, whether they experienced GI symptoms or not, indicates that intestinal permeability changes alone do not account for GI symptom development during running.

## Abbreviations

GI: Gastrointestinal
I-FABP: Intestinal fatty acid-binding protein
LPS: Bacterial lipopolysaccharide
